# Fetal US and MRI in detection of craniospinal anomalies with postnatal correlation: single-center experience

**DOI:** 10.3906/sag-2011-122

**Published:** 2021-06-28

**Authors:** İlker EYÜBOĞLU, Gülseren DİNÇ

**Affiliations:** 1 Department of Radiology, Faculty of Medicine, Karadeniz Technical University, Trabzon Turkey; 2 Department of Obstetrics and Gynecology, Faculty of Medicine, Karadeniz Technical University, Trabzon Turkey

**Keywords:** Fetal MRI, fetal ultrasound, craniospinal malformations, prenatal diagnosis

## Abstract

**Background/aim:**

To reveal the contribution of magnetic resonance imaging (MRI) to ultrasound (US) in prenatal diagnosis of fetal craniospinal anomalies by retrospectively comparing the prenatal and postnatal findings.

**Materials and methods:**

After institutional review board approval, between January 2010 and May 2020, 301 pregnant women, which had a gestational age between 19–37 weeks (mean 26.5 ± 6.1 weeks), diagnosed with cranial and spinal anomalies on fetal US and later on imaged with MRI were evaluated, and in 179 of those cases prenatal imaging findings were compared with postnatal findings.

**Results:**

A total of 191 fetal craniospinal anomalies were detected in 179 pregnant women. MRI and US diagnosis were completely correct in 145 (75.9%) and 112 (58.6%), respectively. Diagnostic performance of MRI was significantly higher than that of the US (p < 0.05). Both prenatal MRI and US findings were concordant with postnatal diagnosis in 53% of the cases. In 28.7% cases, prenatal MRI contributed to US by either changing the wrong US diagnosis (8.9%), demonstration of additional findings (14%), or confirming the suspicious US diagnosis (5.8%).

**Conclusion:**

Due to its high resolution and multiplanar imaging capability, fetal MRI contributes significantly to US in the correct prenatal diagnosis of craniospinal anomalies. This contribution especially is significant in neural tube defects, cortical malformations, and ischemic-hemorrhagic lesions.

## 1. Introduction

Ultrasound (US) is the standard and first-choice method used for fetal anomaly screening. It is a “real-time” imaging modality that is easily available, cheap, easy to use and safe for the fetus since it does not contain ionizing radiation. However, it may be insufficient in complicated malformations due to reasons such as obesity of the mother, unsuitable fetal position, and ossification of cranial bones and oligohydramnios in later gestational weeks [1–3]. In these cases, magnetic resonance imaging (MRI) is frequently used as a complementary method to US [4,5]. Fetal pathologies can be evaluated in more detail with MRI due to its high contrast and spatial resolution and ability to take images in every plane [2,6]. Fetal cranium, spinal cord and related structures are among the areas where fetal MRI is mostly used [4,7,8].

In the present study, we aimed to reveal the contribution of MRI to US in the prenatal diagnosis cranial and spinal anomalies. 

## 2. Materials and methods

Between January 2010 and May 2020, additional fetal MRI was performed on 1028 pregnant women who were found to have fetal anomalies in obstetric US. Craniospinal anomaly was detected with prenatal US and MRI in 301 of 1028 pregnant women. Postnatal data were available in 179 of these cases. Patient data were obtained from the hospital information system, and radiological images from the picture archiving and communication systems (PACS). This retrospective study was approved by the by local ethics committee (2020/298) and the requirement for patient informed consent was waived. Postnatal definitive diagnoses were made by physical examination, inspection, postnatal radiological imaging, autopsy, surgery, pathological examination and/or clinical follow-up. 

### 2.1. Fetal imaging

#### 2.1.1. Obstetric ultrasound

Ultrasonographic examinations were performed by physicians (AC and TA) with at least 5 years of experience in obstetric US with two high resolution US scanners―Voluson 730 expert (General Electric, Waukesha, Wisconsin) or Siemens Sonoline Antares (Siemens Medical Systems, Erlangen, Germany)― with 2–6 MHz transabdominal transducers. All US examinations and cranial and spinal evaluations were performed according to the international society of ultrasound in obstetrics and gynecology (ISUOG) practical guidelines [3,9]. 

Fetal cranial shape, cavum septum pellucidum, falx cerebri, thalami, cerebral hemispheres, ventricles and cerebellum were evaluated in fetal cranial examination. In fetal face examination, orbits, lateral face profile, mouth and lips were examined. Fetal spinal examination was evaluated in longitudinal and axial sections and in terms of bone integrity of the vertebral column at all levels, vertebral anomalies, sacral agenesis, spina bifida, meningocele, meningomyelocele, whether the skin is intact or not and additional cranial, and spinal anomalies [2,3,9]. Only US findings obtained in our center were used in the evaluations. US examinations of cases referred from external centers were repeated in our hospital. More than 95% of MRI examinations were performed within the first week after fetal US.

#### 2.1.2. Fetal magnetic resonance imaging

For fetal neuroimaging, 1.5 Tesla MR unit (Magnetom, Symphony; Siemens, Erlangen, Germany) was used between 2010 and 2015, and 3 Tesla MR unit (Magnetom, Skyra; Siemens Healthcare, Erlangen, Germany) was used between 2015 and 2020. All subjects were examined by using a body phased-array coil. Patients were placed in the supine or lateral decubitus position. Sedation or contrast media were not used.

##### 5T) 2.1.2.1. MRI parameters (1.5T)

Steady state free precession (SSFP) (true fast imaging with steady state precession: true FISP) [TR/TE], 4.9/2.5; matrix, 412 × 512; FA, 80°; NEX, 1; slice thickness, 3 mm; distance factor, 30%) and half Fourier acquisition single shot turbo spin-echo (HASTE) sequence (TR/TE, 4000/86; matrix, 256 × 256; FA, 125°; NEX, 1; slice thickness, 3 mm; distance factor, 30%), T1-weighted spoiled gradient-echo (fast low angle shot: FLASH) (TR/TE, 107/4.8; matrix, 145 × 256; FA, 70°; NEX, 1; slice thickness, 5 mm; distance factor, 30%). Spin-echo, echo-planar diffusion weighted imaging (DWI) (TR/TE, 4000/94; matrix, 128 × 128; NEX, 1; slice thickness, 4 mm; distance factor, 30%, b = 0, 500, 1000).

##### 2.1.2.2. MRI parameters (3T)

T1 volumetric interpolated breath-hold examination (VIBE) [TR/TE], 3.97/1.23; matrix, 195 × 320; FA, 9°; NEX, 1; slice thickness, 3 mm; distance factor, 20%), steady state free precession (SSFP) (true fast imaging with steady state precession: true FISP) [TR/TE], 481/1.87; matrix, 210 × 320; FA, 60°; NEX, 1; slice thickness, 3 mm; distance factor, 20%) and half Fourier acquisition single shot turbo spin-echo (HASTE) sequence (TR/TE, 1200/87; matrix, 256 × 320; FA, 160°; NEX, 1; slice thickness, 3 mm; distance factor, 30%) and spin-echo, echo-planar diffusion weighted imaging (DWI) (TR/TE, 4000/94; matrix, 128 × 128; NEX, 1; slice thickness, 4 mm; distance factor, 30%, b = 0, 1000).

Axial, coronal and sagittal plans were obtained by taking into account the head position of the fetus. The field of view (FOV) was determined according to maternal and fetus sizes.

### 2.2. Evaluation of MR images

All MR images were interpreted in a consensus on MR workstation (Leonardo, Siemens) by two radiologists (S.K. and İ.E.) who experienced in pediatric neuroradiology. Both radiologists were aware of the prenatal US data.

### 2.3. Statistical analysis

Statistical analyses were performed using the SPSS software, v: 27.0 (IBM Corporation, Armonk, NY, USA). Prenatal US and MRI findings were compared in terms of discrepancies and consistencies with each other in the study The diagnoses were classified as totally correct, partially correct, suspected or failed by comparing with final diagnoses and grouped as; 

1. Both US and MRI correct. 

2. MRI correct, US failed. 

3. MRI showed additional findings to US. 

4. MRI confirmed the suspicious US diagnosis.

5. Both US and MRI partially correct.

6. US correct, MRI failed.

7. Both US and MRI were not consistent with postnatal findings.

A marginal homogeneity test was used to compare the diagnostic performance of US and MRI in 191 anomalies. A p-value less than 0.05 (typically ≤ 0.05) was accepted as statistically significant.

## 3. Results

During the ten-year period, there were 301 cases with craniospinal anomalies detected by prenatal US and MRI in our hospital. The gestational age of the pregnant women was between 19–37 weeks (mean 26.5 ± 6.1 weeks). Prenatal diagnosis of 301 fetuses included in the study are summarized in Table 1. Classifying was done based on the major anomaly. The most common anomaly detected by US and MRI, during prenatal period, was ventricular anomalies (32.9%). Asymmetric lateral ventricle enlargement (40.4%) was the most common ventricular anomaly. Posterior fossa anomalies were the second most common anomalies with a rate of 32.2%. Neural tube defects were observed in 12.6% of cases. Myelomeningocele was the most common (31.5%) of them. Midline anomalies were observed in 9.3% of cases during prenatal period. Corpus callosum anomaly constituted 60% of midline anomalies. All of the ischemic-hemorrhagic lesions observed during prenatal period were germinal matrix hemorrhages, and it was 5.6% in our series. Cortical malformations were observed at a rate of 2.7%. Galen vein malformation was detected in 1 (0.4%) case and 4 cases (44%) were Walker–Warburg syndrome. In prenatal period, the diagnosis made by US in 213 (70.8%) cases was also confirmed by MRI. MRI findings were partially compatible with US in 19 (6%) cases. MRI detected additional anomalies in 35 (12%) of cases. 

**Table 1:** Prenatal diagnosis

Prenatal diagnosis	n (%)	Subgroup	
Ventricular anomalies	99 (32.9%)	Asymmetry of the lateral ventricles Isolated ventriculomegaly Isolated hydrocephalus	403128
Posterior fossa anomalies	97 (32.2%)	Isolated cerebellar hypoplasiaDandy-Walker malformationBlake’s pouch cystChiari II malformationMega cisterna magna	81515023
Neural tube defects	38 (12.6%)	Neurenteric cyst, encephalocele IniencephalyMyelocele Myelomeningocele Lipomyelomeningocele Meningocele Tight filum terminale Diastematomyelia Caudal agenesis	4111215851
Midline anomalies and cysts	28 (9.3%)	Isolated agenesis of the corpus callosum HoloprosencephalyArachnoid cyst Connatal cysts	17551
Cortical malformations	8 (2.7%)	SchizencephalyHeterotopiaPolymicrogyriaLyssencephaly	1232
Vascular anomalies	1 (0.4%)	Galen vein malformation	1
Ischaemic-haemorrhagic lesions	17 (5.6%)	Germinal matrix hemorrhage	17
Tumors	4 (1.3%)	Sacrococcygeal teratoma	4
Others	9 (3%)	Walker-Warburg syndrome Dolichocephaly BrachycephalyHypertelorismAnophthalmia	42111

n = number of cases.

There were a total of 179 cases for them postnatal definite diagnoses were available. A total of 191 craniospinal anomalies were detected in these cases. They are summarized in Table 2. Thirteen fetuses with suspected anomalies were diagnosed as normal in postnatal period. 

**Table 2:** Postnatal final diagnosis.

Postnatal diagnosis	n (%)	Subgroup	
Ventricular anomalies	38 (19.9%)	Asymmetry of the lateral ventricles Ventriculomegaly Hydrocephalus /Aqueduct stenosis	8921
Posterior fossa anomalies	58 (30.4%)	Cerebellar hypoplasia/dysplasiaDandy-Walker malformationChiari II malformationMega cisterna magnaRhombencephalosynapsis	464251
Neural tube defects	27 (14.1%)	Neurenteric cyst, encephalocele/cephalocele IniencephalyMyelocele Myelomeningocele Lipomyelomeningocele Meningocele Tight filum terminale (isolated)DiastematomyeliaVertebral segmentation anomalies (isolated)Caudal agenesis	41111121411
Midline anomalies and cysts	26 (13.6%)	Agenesis of the corpus callosum HoloprosencephalyArachnoid cyst Septo-optic dysplasia	18521
Cortical malformations	17 (8.9%)	SchizencephalyHeterotopiaPolymicrogyriaLyssencephalyHemimegaloencephaly	13571
Vascular anomalies	1 (0.5%)	Galen vein malformation	1
Ischaemic-haemorrhagic lesions	12 (6.3%)	Germinal matrix hemorrhagePorencephalic cyst	102
Tumors	5 (2.6%)	Sacrococcygeal teratoma	5
Others	7 (3.7%)	Walker-Warburg syndromeCalcification (Periventricular/parenchymal)	43

n = number of anomalies.

**Figure 1:**
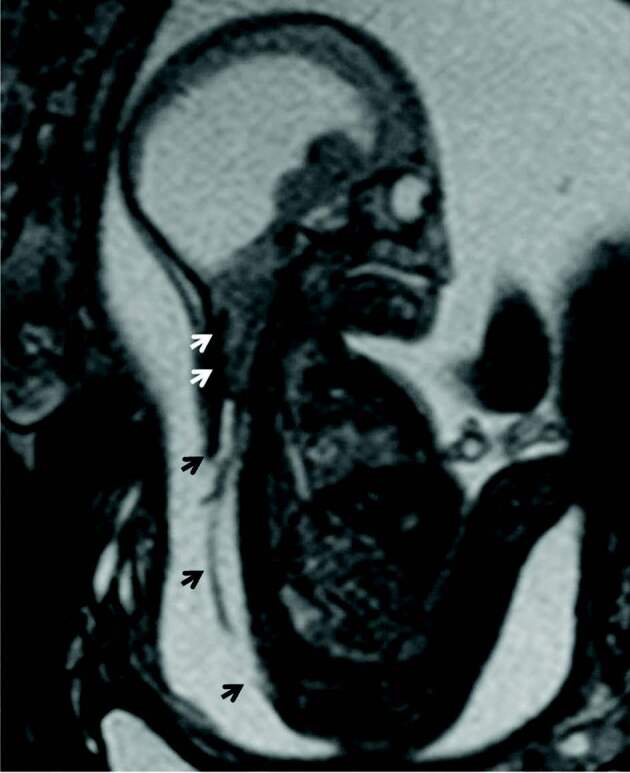
Sagittal T2 HASTE from fetal MR imaging demonstrates severe cerebellar ectopia (white arrows) and large defect in the posterior arch of the thoracic and lumbosacral vertebrae (black arrows). Findings are consistent with Chiari II malformation.

**Figure 2:**
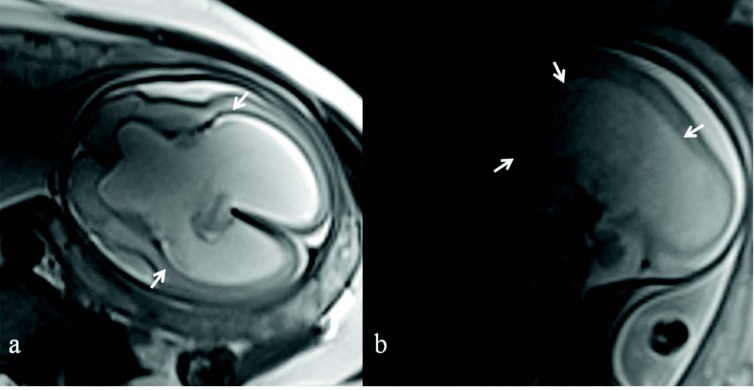
Axial (a) and sagittal (b) T2 HASTE fetal MR images show hydrocephalic dilatation of the lateral ventricles.

**Figure 3:**
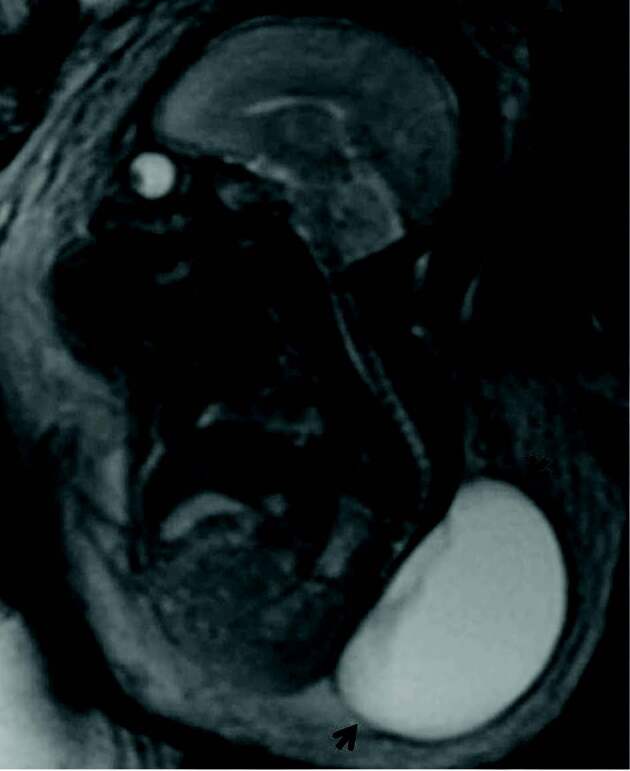
Sagittal T2 SSFSE fetal MR image reveals large lumbosacral myelomeningocele sac (arrows).

**Figure 4:**
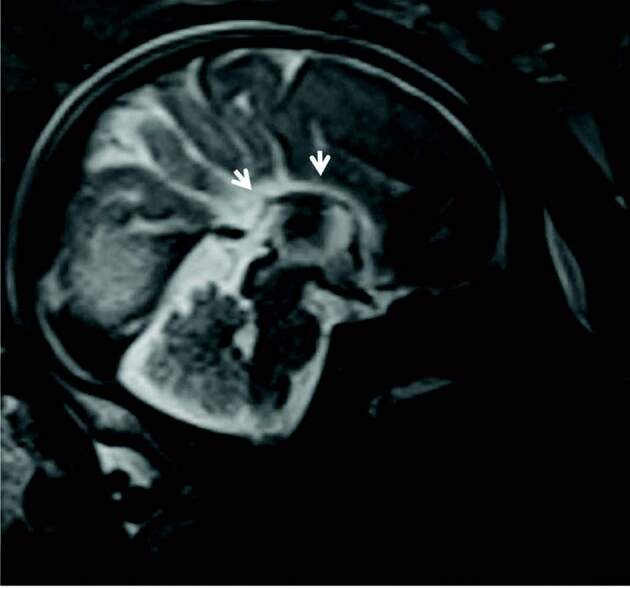
Sagittal T2 SSFSE fetal MR image demonstrates total agenesis of the corpus callosum.

The most common anomaly detected in the postnatal period was posterior fossa anomalies at a rate of 30.4%. Chiari 2 malformation (Figure 1), one of the posterior fossa anomalies, was detected in 72.4%. Ventricular anomalies were observed at a rate of 19.9%. The most common ventricular anomaly in the postnatal period was hydrocephalus associated with aquaduct stenosis (Figure 2a and 2b) and was detected at a rate of 55%. Neural tube defects were observed 14.1% of cases and, myelomeningocele (Figure 3) was the most common (41%) in this group. 72.4% of midline anomalies observed in the postnatal period were corpus callosum agenesis (Figure 4).

US and MRI diagnosis were totally correct in 58.6% and 75.9%, partially correct in 5.2% and 6.3%, suspected in 4.7% and 1.0% and failed in 31.4% and 16.8% of the anomalies, respectively (Table 3). Diagnostic performance of MRI was significantly higher than that of the US (p < 0.05). The comparison of the imaging findings was shown in Table 4.

**Table 3:** US and MRI diagnoses.

	Imaging modality	Number ofanomalies (%)
Totally correct	MRI	145 (75.9 %)
	US	112 (58.6 %)
Partially correct	MRI	12 (6.3%)
	US	10 (5.2%)
Suspected	MRI	2 (1%)
	US	9 (4.7%)
Failed	MRI	32 (16.8%)
	US	60 (31.4%)

US = ultrasonography, MRI = magnetic resonance imaging.

**Table 4:** Comparison of US and MRI diagnoses.

	Number of cases (%)
Both US and MRI correct	101 (53%)
MRI showed additional findings to US	27 (14%)
MRI confirmed the suspicious US diagnosis	11 (5.8%)
MRI correct, US failed	17 (8.9%)
US correct, MRI failed	3 (1.6%)
Both US and MRI were not consistent with postnatal findings	32 (16.7%)

US = ultrasonography, MRI = magnetic resonance imaging.

Prenatal US could not detect spinal anomaly in 5 (33.3%) of cases who were found to have spinal cord or spinal canal anomaly in the postpartum period. MRI detected germinal matrix bleeding in 5 cases with hydrocephalus and contributed to the differential diagnosis. Eight cases, diagnosed with mild ventriculomegaly on prenatal US and MRI, were normal on postnatal control MRIs. MRI made the correct diagnosis in 11 of 17 fetuses with cortical malformation. US was able to make the correct diagnosis in 6 cases with cortical malformation. Sacrococcygeal teratoma in 3 cases misdiagnosed as sacral meningomyelocele on prenatal US and MRI.

Among the ventricular anomalies, 3 (25%) of 12 aqueduct stenosis cases were not diagnosed correctly by US and 1 (8.3%) by MRI. In one case, both MRI and US misdiagnosed.

Both US and MRI made the correct diagnosis in all cases with Chiari II and Dandy–Walker malformation among posterior fossa anomalies. US was reported as normal in 2 (50%) of 4 cases of cerebellar dysplasia/hypoplasia. MRI could not make a correct diagnosis in 1 (25%) case with cerebellar hypoplasia. Both US and MRI made the correct diagnosis of all 11 cases with isolated meningomyelocele, which were the most common neural tube defects.

## 4. Discussion

In our current study, we evaluated retrospectively the efficiency of US and MRI, performed in the prenatal period, in detecting fetal craniospinal anomaly by comparing with postnatal definite diagnoses. MRI and US confidently diagnosed 145 (75.9%) and 112 (58.6%) of the total 191 anomalies, respectively. MRI made a valuable contribution to US in 28.7% of the cases.

Although obstetric US is the first preferred method in fetal anomaly screening, MRI is frequently used as an auxiliary method to US in evaluating fetal anatomy and pathologies in the last 20 years due to its multiplanar imaging capacity and excellent soft tissue contrast resolution [2,4,6,10]. Craniospinal anomalies are the most important area where MRI contributes to US. Therefore, craniospinal anomalies constitute approximately 80% of fetal MRI examinations. US is inadequate in characterizing the craniospinal malformations due to reasons such as unsuitable fetal position, obesity of the mother, and low amount of amniotic fluid [2,4,8]. Sensitivity of US in brain anomalies was reported to be 88% [4]. In a meta-analysis of 27 studies evaluating fetal brain MRI and US diagnoses, with a total of 1184 cases, fetal MRI contributed to US in 23% of the cases. In the same study, MRI provided correct diagnosis in 8% of cases where US was failed [11]. Similarly, in 9.5% of our cases US failed while MRI was correct. In our study, US failed to diagnose 31.4% of the 191 anomalies. The main anomalies, US failed to diagnose, were neural tube defects, cortical malformations, and ischemic-hemorrhagic lesions. 

The most common indication for fetal brain MRI is ventriculomegaly. 40% of fetal brain and spinal imaging is performed due to ventriculomegaly. Classically a ventricular atrium width of more than 10 mm is considered pathological. Ventriculomegaly carries the risk of impairment and delay in brain development, chromosomal abnormality, and hydrocephalus in early fetal period [4,12–14]. In many cases, mild ventriculomegaly resolves in the postnatal period without creating a clinical problem [5,15]. In our study, postnatal MRIs of 8 cases, whose prenatal US and MRI reported as ventriculomegaly, were normal. Hydrocephalus associated with aquaduct stenosis was the most common (55%) ventricular anomaly observed in the postnatal period. The most common underlying causes of ventriculomegaly were reported as aquaduct stenosis, intracranial bleeding, neural tube defects, corpus callosum agenesis, and congenital infections in the literature [4]. In a prospective randomized study, 17% of cases were found to have ventriculomegaly in fetal MRI. In this study, corpus callosum agenesis was the most common cause of ventriculomegaly [12].

The corpus callosum is the main structure that provides the interhemispheric connection in the brain and it develops between 7–20 weeks of gestation. Destructive events, congenital metabolic and genetic disorders observed during this period may cause corpus callosum anomalies [5,16]. The prevalence of corpus callosum anomalies is approximately 1.8 per 10,000 live births and is mostly associated with other central nervous system malformations such as ventriculomegaly, holoprosencephaly, and Dandy–Walker malformation and midline defects [4,17]. Delayed speech, social behavior and nutrition disorders, hyperactivity, attention disorders, epilepsy, and mental retardation might be seen in these cases during the childhood. However, isolated corpus callosum agenesis is asymptomatic in the postnatal period in 85% of cases and has a good prognosis [5,18]. The sensitivity of prenatal MRI in the diagnosis of midline anomalies (corpus callosum agenesis and cavum septum pellucidum) is reported around 90% [4]. In the review of Sotiriadis et al. [19] covering 132 cases in 16 studies, MRI detected additional cerebral abnormalities in 22.5% of apparently isolated agenesis of the corpus callosum cases. In our study, midline anomalies were found with a rate of 13.6% in the postnatal period and the majority of midline anomalies was corpus callosum agenesis. In our study, the main anomalies accompanying corpus callosum agenesis were cortical malformations. Five of the 18 cases with corpus callosum agenesis had cortical malformation.

Dandy-Walker malformation, arachnoid cyst, holoprosencephaly, and cortical malformations are other common indications of fetal MRI. Cortical malformations are associated with various gyral developmental disorders. The most common known gyral developmental disorders or migration anomalies are polymychrogyria, lissencephaly, pachygyria, schizencephaly, and heterotopia [4,5]. In our series, cortical malformations were detected at a rate of 8.9% in the postnatal period. Lissencephaly was the most common cortical malformation (41%). Lissencephaly or agyria is characterized by the absence of total cerebral gyri and sulci and is the most severe form of migration anomalies [4,20]. It was reported that the most appropriate fetal MRI time for the diagnosis of cortical malformations is between 28 and 32 weeks of gestation [4,21]. The sensitivity of fetal MRI in the diagnosis of cortical anomaly is reported to be 70%–95% [4,10,21]. In the current study, MRI and US diagnosed 11 (64.7%) and 6 (35.3%) of the cortical malformations, respectively.

Posterior fossa anomalies include cerebellum and brainstem lesions. Due to ossification of fetal cranial bones in the last trimester of pregnancy, it becomes difficult to evaluate the posterior fossa with US. In the prenatal period, together with ventricular anomalies and posterior fossa anomalies were one of the most common anomalies (32.9% and 32.2%, respectively) detected in our series. The most common indications of fetal MRI in the posterior fossa were suspicion of Chiari II malformation, mega sisterna magna, Dandy Walker malformation, and cerebellar hypoplasia-dysplasia in our study similar to reported in the literature [4,5,22]. 

In our study, both MRI and US correctly diagnosed all cases with Chiari II malformations. Spinal canal, spinal cord, bony vertebral structures, extradural distances, skin and subcutaneous fat tissues can be evaluated very well with MRI [5,8]. Neural tube defects include the group of congenital anomalies involving the spinal cord in the early gestational weeks (3rd and 5th gestational weeks) with an incidence of around 1–2 per 1000 [4,5,23]. Neural tube defects are classified in several ways: open neural tube defects (myelocele and myelomeningocele), closed or skin-covered neural tube defects (meningocele, lipomeningomyelocele, lipomyelosis), those not associated with subcutaneous mass (split cord, neuroenteric cyst syndrome, or caudal regression, tight filum terminal, and dermal sinus). The most common spinal anomaly is myelomeningocele and it is most commonly observed in the lumbosacral region [4,5,8]. In our study, neural tube defects were observed with a rate of 12.6% in prenatal evaluation and 14.1% in postnatal evaluation. Among the neural tube defects, the most common anomaly in both prenatal and postnatal periods was myelomeningocele (31.5% and 41%, respectively). 

There were some limitations of the present study. One of the main limitations was that only fetuses with suspected fetal anomaly on US were evaluated by MRI. This may have increased the contribution of MRI in the diagnosis of fetal anomaly. The second limitation was that radiologists, evaluating the MRI, were not blind to the US. Thirdly, inter-observer variability was not evaluated in the present study. Although US and MRI examinations are performed by experienced radiologists, findings may show intra and inter-observer variability. Finally, the use of different MRI and ultrasound devices for diagnosis may affect the accuracy of prenatal diagnosis.

As a result, we have documented that MRI has an important complementary role and adds important diagnostic information to US in the prenatal diagnosis of fetal craniospinal anomalies. In approximately 10% of cases, only MRI made the correct prenatal diagnosis. In about 20% of cases, MRI contributed to US, either by additional diagnosis or by confirmation of suspicious findings. The main contribution of MRI to US was in the spinal cord anomaly including diastematomyelia and tight filum terminale, cortical malformations and germinal matrix hemorrhage. On the other hand, prenatal MRI and US failed in 14.6% of the cases, which were mainly mild ventriculomegaly and sacrococcygeal masses.

## References

[ref1] (2004). Routine screening for fetal anomalies: expectations. Obstetrics and Gynecology Clinics of North America.

[ref2] (2012). Contribution of MRI to ultrasound in the diagnosis of fetal anomalies. Journal of Magnetic Resonance Imaging.

[ref3] (2011). ISUOG Clinical Standards Committee. Practice guidelines for performance of the routine mid-trimester fetal ultrasound scan. The Ultrasound in Obstetrics and Gynecology.

[ref4] (2017). Fetal MRI of the central nervous system: state-of-the-art. European Journal of Radiology.

[ref5] (2003). Magnetic resonance imaging and ultrasound in the assessment of the fetal central nervous system. Journal of Perinatal Medicine.

[ref6] (2012). MRI: is there a role in obstetrics? Clinical Obstetrics. Gynecology.

[ref7] (2016). Novel Classification and Contribution to Sonography. Ultraschall in der Medizin.

[ref8] (2011). Magnetic resonance imaging in the evaluation of the fetal spinal canal contents. Brain and Development.

[ref9] (2007). Gynecology Education Committee. Sonographic examination of the fetal central nervous system: guidelines for performing the ‘basic examination’ and the ‘fetal neurosonogram’. The Ultrasound in Obstetrics and Gynecology.

[ref10] (2006). Fetal magnetic resonance imaging. Current Opinion in Obstetrics and Gynecology.

[ref11] (2016). Added value of fetal MRI in fetuses with suspected brain abnormalities on neurosonography: a systematic review and meta-analysis. The Journal of Maternal-Fetal and Neonatal Medicine.

[ref12] (2012). Is fetal magnetic resonance imaging indicated when ultrasound isolated mild ventriculomegaly is present in pregnancies with no risk factors?. Prenatal Diagnosis.

[ref13] (1994). Isolated mild fetal cerebral ventriculomegaly: clinical course and outcome. Radiology.

[ref14] (2007). Postnatal clinical and imaging follow-up of infants with prenatal isolated mild ventriculomegaly: a series of 101 cases. Pediatric Radiology.

[ref15] (1999). The clinical significance of fetal isolated cerebral borderline ventriculomegaly. Report of 31 cases and review of the literature. Ultrasound in Obstetrics and Gynecology.

[ref16] (1994). Prognostic indicators in the prenatal diagnosis of agenesis of corpus callosum. American Journal of Obstetrics and Gynecology.

[ref17] (2008). Agenesis of the corpus callosum in California 1983-2003: a population-based study. American Journal of Medical Genetics Part A.

[ref18] (2001). Outcome in prenatally diagnosed fetal agenesis of the corpus callosum. Fetal Diagnosis and Therapy.

[ref19] (2012). Neurodevelopment after prenatal diagnosis of isolated agenesis of the corpus callosum: an integrative review. American Journal of Obstetrics and Gynecology.

[ref20] (1991). The agyria-pachygyria complex: a spectrum of cortical malformations. Brain and Development.

[ref21] (2012). Malformations of cortical development: diagnostic accuracy of fetal MR imaging. Radiology.

[ref22] (2012). Magnetic resonance imaging of the fetal central nervous system. Seminars in Ultrasound, CT and MRI.

[ref23] (2003). Epidemiology of neural tube defects. Epilepsia.

